# Twin RNA Polymerase–Associated Proteins Control Virulence Gene Expression in Francisella tularensis

**DOI:** 10.1371/journal.ppat.0030084

**Published:** 2007-06-15

**Authors:** James C Charity, Michelle M Costante-Hamm, Emmy L Balon, Dana H Boyd, Eric J Rubin, Simon L Dove

**Affiliations:** 1 Division of Infectious Diseases, Children's Hospital, Harvard Medical School, Boston, Massachusetts, United States of America; 2 Department of Immunology and Infectious Diseases, Harvard School of Public Health, Boston, Massachusetts, United States of America; 3 Department of Microbiology and Molecular Genetics, Harvard Medical School, Boston, Massachusetts, United States of America; University of Chicago, United States of America

## Abstract

The MglA protein is the only known regulator of virulence gene expression in *Francisella tularensis,* yet it is unclear how it functions. F. tularensis also contains an MglA-like protein called SspA. Here, we show that MglA and SspA cooperate with one another to control virulence gene expression in F. tularensis. Using a directed proteomic approach, we show that both MglA and SspA associate with RNA polymerase (RNAP) in *F. tularensis,* and that SspA is required for MglA to associate with RNAP. Furthermore, bacterial two-hybrid and biochemical assays indicate that MglA and SspA interact with one another directly. Finally, through genome-wide expression analyses, we demonstrate that MglA and SspA regulate the same set of genes. Our results suggest that a complex involving both MglA and SspA associates with RNAP to positively control virulence gene expression in F. tularensis. The F. tularensis genome is unusual in that it contains two genes encoding different α subunits of RNAP, and we show here that these two α subunits are incorporated into RNAP. Thus, as well as identifying SspA as a second critical regulator of virulence gene expression in *F. tularensis,* our findings provide a framework for understanding the mechanistic basis for virulence gene control in a bacterium whose transcription apparatus is unique.

## Author Summary

The Gram-negative bacterium Francisella tularensis is an intracellular pathogen and the causative agent of tularemia. In *F. tularensis,* the MglA protein is the only known regulator of virulence genes that are important for intracellular survival, yet it is not known how MglA functions. F. tularensis also contains an MglA-like protein called SspA whose function is not known. In this study, we show that both MglA and SspA associate with RNA polymerase (RNAP) and positively regulate virulence gene expression in F. tularensis. Our study provides evidence that MglA and SspA interact with one another directly and that the association of MglA with RNAP is dependent on the presence of SspA. We also show that, unlike RNAP from any other bacterium, RNAP from F. tularensis contains two distinct α subunits. Given the fundamental roles the α subunit plays in transcription regulation, this may have far-reaching implications for how gene expression is controlled in F. tularensis. Our study therefore uncovers a new critical regulator of virulence gene expression in F. tularensis (SspA), provides mechanistic insight into how MglA and SspA cooperate to control virulence gene expression, and reveals that the F. tularensis transcription machinery has an unusual composition.

## Introduction

Francisella tularensis is a Gram-negative, facultative intracellular pathogen and the aetiological agent of tularemia, a disease that can be fatal in humans. Although outbreaks of tularemia are thought to be rare, infections caused by F. tularensis have become a public health issue because of the potential for using this organism as a bioweapon. As few as ten organisms can constitute an infectious dose, and the pneumonic form of tularemia, which has a particularly high mortality rate, can be acquired when the organism is aerosolized [1,2]. Relatively little is known regarding the molecular mechanisms of F. tularensis pathogenesis, in part because of the genetic intractability of this organism.

Evidence suggests that the pathogenicity of F. tularensis depends on its ability to survive and replicate within macrophages. However, only a handful of genes that are required for intramacrophage survival have been identified [2–4]. One of these encodes a putative transcription regulator called macrophage growth locus A (MglA) that is responsible for controlling the expression of multiple virulence factors that are themselves required for survival within macrophages [3,5,6]. In particular, in F. tularensis subspecies *novicida,* MglA positively regulates the expression of multiple virulence genes, some of which are located on a pathogenicity island [5–7]. MglA also regulates the expression of many other genes that may or may not be implicated in virulence [5,6]. However, the mechanism of this MglA-dependent regulation is unknown.

MglA is an ortholog of the stringent starvation protein A (SspA) from Escherichia coli (the two proteins share 21% identity and 34% similarity at the primary amino acid level). In *E. coli,* SspA is a RNA polymerase (RNAP)–associated protein that is thought to regulate the expression of a subset of genes under conditions of stress [8,9]. SspA in E. coli is also essential for the lytic growth of bacteriophage P1 [10]. Specifically, Hansen and colleagues have shown that E. coli SspA functions as an activator of P1 late gene expression, acting in concert with a phage-encoded transcription activator called Lpa [11]. SspA homologs have also been found in a number of pathogens other than *F. tularensis,* and several of these homologs appear to play important roles in virulence gene regulation [12–14].

Recently, the genomes of two different strains of F. tularensis have been sequenced ([15]; GenBank accession number NC_007880). One of these strains is Schu S4, a highly virulent strain of subspecies *tularensis,* and the other is the live vaccine strain (LVS), an attenuated derivative of one of the subspecies *holarctica* strains. The genome sequences reveal that F. tularensis contains an MglA-like protein, which has been annotated as SspA [15]. In addition, the sequences reveal that, unlike any other bacterial genome sequenced to date, the F. tularensis genome contains two genes, designated *rpoA1* (or FTT0350 in the annotated Schu S4 genome sequence) and *rpoA2* (FTT1442), that encode different α subunits of RNAP. The α subunit of bacterial RNAP forms a dimer in the RNAP complex (subunit composition α_2_ββ′ωσ). The two putative α subunits in F. tularensis differ from one another in regions that are predicted to be critical for dimer formation, promoter recognition, and activator interaction. Given the fundamental roles the α subunit plays in transcription regulation [16], this may have far-reaching implications for how gene expression is controlled in F. tularensis.

Here, we show that both MglA and SspA associate with RNAP and positively control virulence gene expression in F. tularensis. Furthermore, we present evidence that the ability of MglA to control virulence gene expression is linked to its ability to associate with RNAP. We also show that the two distinct α subunits encoded by the F. tularensis genome are both incorporated into RNAP. Our results provide a framework for understanding the mechanistic basis for virulence gene control in this organism.

## Results

### MglA and SspA Associate with RNAP in F. tularensis

**Figure 1:**
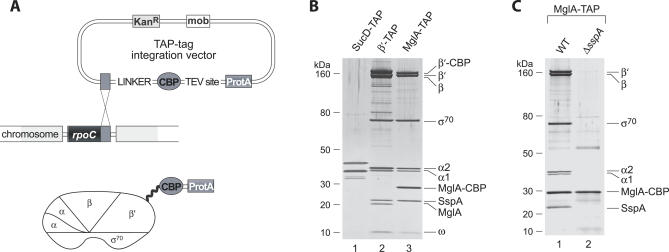
MglA and SspA Are Associated with RNAP in F. tularensis (A) Schematic representation of the TAP-tag integration vector and its use in making β′-TAP. The calmodulin binding peptide (CBP), protein A moieties (ProtA), and TEV cleavage site that constitute the TAP-tag are shown [17], together with the kanamycin resistance determinant (Kan^R^) and the mobilization region (mob). (B) SDS-PAGE analysis of proteins that co-purify with SucD-TAP, β′-TAP, and MglA-TAP from F. tularensis. Protein complexes were tandem affinity purified, electrophoresed on a 4%–12% Bis-Tris NuPAGE gel, and stained with silver. Lane 1, proteins purified from strain LVS SucD-TAP. Lane 2, proteins purified from strain LVS β′-TAP. Lane 3, proteins purified from strain LVS MglA-TAP. MglA, SspA, and subunits of RNAP are indicated together with the β′-CBP and MglA-CBP fusions that result following cleavage of each corresponding TAP fusion with TEV protease. MS/MS analyses revealed that many of the proteins that run between the β and σ^70^ subunits of RNAP (in lane 2) are breakdown products of the β subunit (unpublished data). (C) SDS-PAGE analysis of proteins that co-purify with MglA-TAP in a wild-type (WT, lane 1) or in a Δ*sspA* mutant background (lane 2). Protein complexes were tandem affinity purified, electrophoresed on a 4%–12% Bis-Tris NuPAGE gel, and stained with silver. Lane 1, proteins purified from strain LVS MglA-TAP. Lane 2, proteins purified from strain LVS Δ*sspA* MglA-TAP. Molecular weights are indicated on the left.

To address the question of whether MglA and SspA are associated with RNAP in *F. tularensis,* we took a directed proteomic approach in which we affinity purified RNAP from F. tularensis and identified candidate RNAP-associated proteins by tandem mass spectrometry (MS/MS). To facilitate affinity purification of F. tularensis RNAP, we adapted the tandem affinity purification (TAP) strategy [17] for use in F. tularensis. Using the integration vector depicted in Figure 1A, we constructed a strain of F. tularensis LVS in which the native chromosomal copy of the *rpoC* gene (encoding the β′ subunit of RNAP) has been modified to encode a TAP-tagged form of β′ (β′-TAP). The resulting strain (LVS β′-TAP) thus synthesizes, at native levels, β′ with a TAP-tag fused to its C-terminus. As a control we constructed, in a similar fashion to the LVS β′-TAP strain, a strain that synthesizes SucD (a subunit of the succinyl CoA synthetase complex) with a TAP-tag fused to its C-terminus (LVS SucD-TAP). Lysates were made from cells of the LVS SucD-TAP strain and cells of the LVS β′-TAP strain. Proteins were then purified by TAP essentially as described [18], separated by SDS-PAGE, and stained with silver. Two proteins with apparent molecular weights of ~20 kDa were found that specifically co-purified with β′ (Figure 1B). Bands corresponding to these proteins were excised from the gel, and, following in-gel digestion with trypsin, nanoelectrospray MS/MS was used to identify the proteins as MglA and SspA (unpublished data). Among the proteins that appear to specifically co-purify with β′, one had an apparent molecular weight of ~70 kDa, two had apparent molecular weights of ~40 kDa, and one had an apparent molecular weight of ~10 kDa (Figure 1B). Bands corresponding to these proteins were excised from the gel, and, following in-gel digestion with trypsin, nanoelectrospray MS/MS was used to determine the identity of each protein. Accordingly, the ~70- and ~10-kDa proteins were found to be the σ^70^ and ω subunits of RNAP, respectively, and the two ~40-kDa proteins were found to be α1 and α2, the products of the *rpoA1* and *rpoA2* genes, respectively (unpublished data). Therefore, F. tularensis RNAP contains two distinct α subunits and associates with both MglA and SspA.

### The Association of MglA with RNAP Is Dependent upon SspA

We reasoned that if MglA were associated with RNAP in *F. tularensis,* then RNAP would be expected to co-purify with TAP-tagged MglA. To test this prediction, we constructed a strain of LVS that synthesized MglA with a TAP-tag fused to its C-terminus (LVS MglA-TAP). Figure 1B shows the results following TAP of MglA from this strain. In support of the hypothesis that MglA associates with RNAP in *F. tularensis,* subunits of RNAP (including α1, α2, β, and β′) co-purified with MglA. Furthermore, SspA also co-purified with MglA. If MglA and SspA were to interact with RNAP independently of one another, then SspA might not be expected to co-purify with MglA-TAP. In particular, complexes containing RNAP and MglA would be expected to be distinct from those containing RNAP and SspA if MglA and SspA competed for the same binding site on RNAP. It is of course possible that there is more than one contact site for MglA and/or SspA on RNAP. However, because SspA from Yersinia pestis is a dimer [19], one plausible explanation for our results is that MglA and SspA physically interact with one another and associate with RNAP as a heterodimer. An alternative possibility is that MglA might form two distinct complexes, one with SspA, and another with RNAP; in this case, the SspA that co-purifies with β′-TAP (Figure 1B, lane 2) would represent SspA-RNAP complexes that are distinct from MglA-RNAP complexes. In an attempt to distinguish between the possibilities that MglA and SspA form a single complex or separate complexes with RNAP, we constructed a strain that synthesized MglA-TAP and carried an in-frame deletion of *sspA* (LVS Δ*sspA* MglA-TAP). Figure 1C shows the results following TAP of MglA-TAP from cells of this strain. In the absence of SspA, MglA fails to co-purify with RNAP. Our findings are therefore consistent with the hypothesis that MglA and SspA are associated with one another in F. tularensis and that the resulting MglA–SspA complex associates with RNAP.

### MglA and SspA Interact with One Another Directly

**Figure 2:**
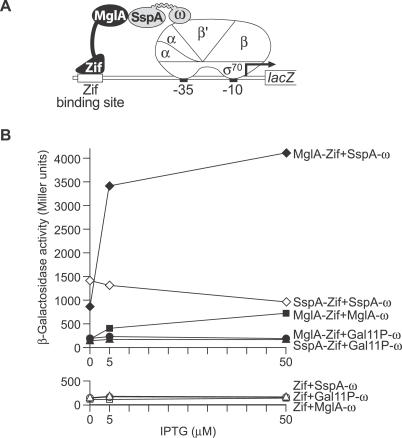
Bacterial Two-Hybrid Analysis of Protein–Protein Interactions Involving MglA and SspA (A) Schematic representation of the two-hybrid system. Contact between MglA and SspA fused, respectively, to Zif and to the ω subunit of E. coli RNAP activates transcription from the test promoter, driving expression of *lacZ*. The diagram depicts test promoter p*lac*Zif1–61, which bears a Zif binding site centered 61 bp upstream of the transcription start site of the *lac* core promoter (whose −10 and −35 elements are indicated). In E. coli strain KDZif1ΔZ, this test promoter is linked to *lacZ* on an F′ episome. (B) Transcription activation by MglA-Zif in the presence of the SspA-ω or MglA-ω fusion proteins, and by SspA-Zif in the presence of the SspA-ω fusion protein. KDZif1ΔZ cells harboring compatible plasmids directing the synthesis of the indicated proteins were grown in the presence of different concentrations of IPTG and assayed for β-galactosidase activity.

In order to test explicitly whether or not MglA and SspA can interact with one another directly, we used a bacterial two-hybrid assay configured to permit the detection of both dimeric and higher order complexes. This two-hybrid assay is based on the finding that any sufficiently strong interaction between two proteins can activate transcription in *E. coli,* provided that one of the interacting proteins is tethered to the DNA by a DNA-binding protein and the other is tethered to a subunit of E. coli RNAP [20,21]. Specifically, contact between a protein (or protein domain) fused to the ω subunit of E. coli RNAP and another protein fused to a zinc finger DNA-binding protein (referred to as Zif) activates transcription of a *lacZ* reporter gene (with an upstream Zif binding site) in E. coli cells (Figure 2A) [22]. Because Zif binds its cognate recognition site as a monomer, and because the ω subunit is monomeric in the RNAP holoenzyme complex, this configuration of the assay is ideally suited to detecting interactions between two protein monomers (i.e., dimer formation).

Our strategy for determining whether MglA can interact with SspA (thus potentially forming a heterodimer) involved the use of two fusion proteins, one comprising MglA fused to Zif and the other comprising F. tularensis SspA fused to the ω subunit of E. coli RNAP (Figure 2A). Accordingly, we fused full-length MglA (residues 1–205) to the N-terminus of Zif, and we fused full-length SspA (residues 1–210) to the N-terminus of ω. We then tested whether the MglA-Zif fusion protein could activate transcription from an appropriate test promoter in cells containing the SspA-ω fusion protein. Plasmids expressing the MglA-Zif and SspA-ω fusion proteins were introduced into E. coli strain KDZif1ΔZ, which harbors a test promoter linked to *lacZ* on an F′ episome (Figure 2A; [22]). In support of the idea that MglA and SspA interact with one another directly, the MglA-Zif fusion protein activated transcription of the reporter gene strongly (up to ~22-fold) in cells containing the SspA-ω fusion protein, whereas Zif (without the fused MglA moiety) did not (Figure 2B). The observed activation was dependent upon the ability of the ω moiety of the SspA-ω fusion to interact with E. coli RNAP (unpublished data). Another control revealed that MglA-Zif did not activate transcription from the test promoter in the presence of the unrelated Gal11P-ω fusion protein (Figure 2B).

Given that MglA and SspA are similar to one another at the primary amino acid level, we sought to determine whether MglA or SspA (or both) could also form homomeric complexes. We therefore constructed two additional fusion proteins, one in which full-length MglA was fused to the N-terminus of ω, and another in which full-length F. tularensis SspA was fused to the N-terminus of Zif. Results depicted in Figure 2B show that specific interactions were also detected between the MglA-Zif and MglA-ω fusion proteins, as well as between the SspA-Zif and SspA-ω fusion proteins. Our findings suggest that MglA and SspA can form both heteromeric and homomeric complexes.

**Figure 3:**
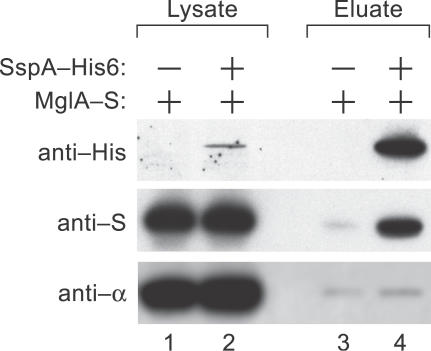
MglA Co-Purifies with His-Tagged SspA Immunoblot analyses of proteins co-purifying with SspA-His6. Cell lysates of E. coli containing MglA-S and SspA (i.e., without the His-tag) or MglA-S and SspA-His6 were incubated with a metal chelate affinity resin. Following washing, proteins specifically bound to the resin were eluted with imidazole. Cell lysates and eluted proteins were separated on a 4%–12% Bis-Tris NuPAGE gel and analyzed by immunoblotting using antibodies that recognize the His-tag on SspA-His6 (anti-His; upper panel), the S-tag on MglA-S (anti-S; middle panel), and the α subunit of E. coli RNAP (anti-α; lower panel). Lane 1, lysate containing MglA-S and SspA. Lane 2, lysate containing MglA-S and SspA-His6. Lane 3, proteins purified from the lysate containing MglA-S and SspA. Lane 4, proteins purified from the lysate containing MglA-S and SspA-His6.

As an additional test of whether MglA and SspA interact with one another to form a heteromeric complex, we co-expressed genes encoding S-tagged MglA (MglA-S) and His-tagged SspA (SspA-His6) in E. coli and asked whether the two proteins co-purified with one another following metal chelate affinity chromatography. Specifically, we made a cell lysate from *E. coli,* synthesizing both SspA-His6 and MglA-S. We then applied the lysate to a metal chelate affinity resin to capture SspA-His6 and any associated proteins, washed the resin, and eluted proteins that were specifically bound to the resin with imidazole. As a control, we treated an E. coli cell lysate containing both non-tagged SspA and MglA-S in an identical manner. (It was important to use non-tagged SspA as a control, rather than no SspA, because MglA-S is insoluble when expressed alone, at least under the conditions of our experiment.) Immunoblot analyses revealed that equal amounts of soluble MglA-S were present in both lysates and that MglA-S co-purified with SspA-His6 (Figure 3). Furthermore, immunoblotting with an antibody that recognizes the α subunit of E. coli RNAP revealed that the association between MglA-S and SspA-His6 was not mediated indirectly through interactions with the E. coli RNAP complex (Figure 3). These findings provide additional evidence that MglA and SspA interact with one another directly.

### Both MglA and SspA Regulate Virulence Gene Expression in F. tularensis

Previous studies revealed that MglA positively regulates expression of the *pdpD, iglA, iglC, iglD,* and *pdpA* genes in F. tularensis subspecies *novicida* (and of these, *iglA, iglC,* and *pdpA* have been implicated in intramacrophage survival and virulence) [4,5,7]. These genes are located on a pathogenicity island [7]. In subspecies *novicida* there is only one copy of this island [7], whereas in the highly virulent Schu S4 strain (subspecies *tularensis*), there are two identical copies of the pathogenicity island [15]. Similarly, the attenuated strain LVS (a derivative of one of the subspecies *holarctica* strains), contains two copies of the pathogenicity island. We have shown that in LVS both MglA and SspA associate with RNAP and that the association of MglA with RNAP is dependent upon SspA. We therefore reasoned that if MglA controls virulence gene expression through its association with RNAP, then the absence of SspA in an *sspA* mutant strain should result in decreased levels of virulence gene expression. That is to say, we would expect that both MglA and SspA are required for virulence gene expression. To compare the effect on virulence gene expression of removing either MglA or SspA in LVS, we constructed mutant strains of LVS carrying in-frame deletions of either the *mglA* gene (LVS Δ*mglA*) or the *sspA* gene (LVS Δ*sspA*). Because both Δ*mglA* and Δ*sspA* mutant cells had a growth defect compared to wild-type LVS cells (culture doubling times of ~171, ~182, and ~147 min, respectively, under the conditions of our experiment), we also used, as a control, cells of a mutant containing a mariner transposon in the gene FTL_0951 (LVS::*FTL0951*); this strain has a more severe growth defect than either the Δ*mglA* or Δ*sspA* mutant strain (with a corresponding culture doubling time of ~285 min). Cells of the wild-type LVS strain, cells of the LVS Δ*mglA* mutant strain, cells of the LVS Δ*sspA* mutant strain, and cells of the control strain LVS::*FTL0951* were grown to mid-log (where, in the wild-type strain, the *mglA* and *sspA* genes are highly expressed; see Figure S1), total RNA was isolated, and expression of the *iglA, iglC,* and *pdpA* virulence genes in each of the four strains was measured using quantitative real-time reverse transcriptase (RT)–PCR.

**Figure 4:**
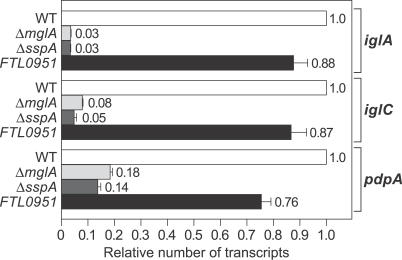
Both MglA and SspA Positively Control Virulence Gene Expression in F. tularensis Quantitative RT-PCR analysis of *iglA, iglC,* and *pdpA* transcript levels in wild-type (WT), Δ*mglA,* Δ*sspA,* and *FTL0951* mutant backgrounds. Transcripts were normalized to *tul4* whose expression is not influenced by MglA or SspA; compared to cells of the wild-type strain, cells of the Δ*mglA* mutant had 0.91 times (with an SE of 0.04) the number of *tul4* transcripts, as determined by quantitative RT-PCR. Similarly, compared to cells of the wild-type strain, the Δ*sspA,* and *FTL0951* mutants had 1.02 times (SE = 0.12) and 1.47 times (SE = 0.09) the number of *tul4* transcripts, respectively.

The results depicted in Figure 4 show drastically reduced amounts of the *iglA, iglC,* and *pdpA* transcripts in both LVS Δ*mglA* mutant cells and LVS Δ*sspA* mutant cells when compared to LVS wild-type cells. Complementation of the LVS Δ*mglA* and LVS Δ*sspA* mutant cells with plasmids expressing either the *mglA* or the *sspA* gene, respectively, restored the amounts of *iglA, iglC,* and *pdpA* transcripts to near wild-type levels (Figure S2). Moreover, the effects of the *mglA* and *sspA* deletions on virulence gene expression were not simply due to the effect of these deletions on growth rate; only a modest reduction in the amounts of the *iglA, iglC,* and *pdpA* transcripts were evident in cells of the slow growing LVS::*FTL0951* mutant when compared to LVS wild-type cells (Figure 4). Thus, both MglA and SspA appear to positively control expression of the *iglA, iglC,* and *pdpA* virulence genes in LVS, though we cannot exclude the possibility of indirect effects. Because we showed that the association of MglA with RNAP is dependent on SspA (Figure 1C), we infer that MglA controls virulence gene expression through its association with RNAP. Taken together, our findings suggest that MglA and SspA interact with one another directly to form a complex that associates with RNAP to positively control expression of the *iglA, iglC,* and *pdpA* virulence genes located on the F. tularensis pathogenicity island.

### MglA and SspA Co-Regulate the Same Set of Genes in F. tularensis

**Figure 5:**
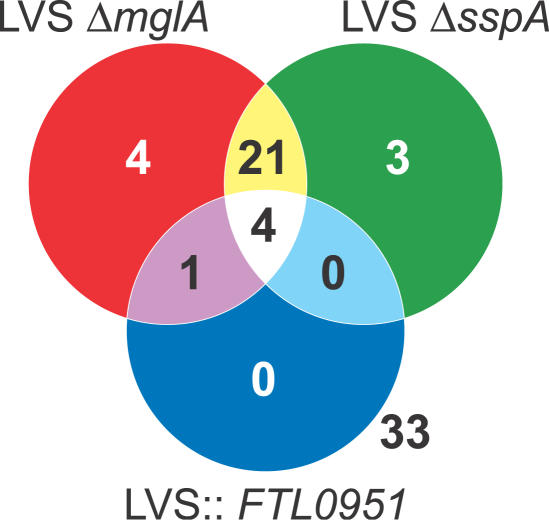
MglA and SspA Control the Expression of a Similar set of Genes Venn diagram representation of the overlap between genes controlled by MglA, SspA, or growth rate. Each circle represents those genes whose expression was altered by a factor of 3 or more (*p* < 0.01) in the corresponding mutant strain compared to wild-type, and whose expression altered by a factor of 3 or more (*p* < 0.01) in the other mutant background.

Recently, DNA microarray analyses have revealed that in subspecies *novicida,* MglA controls the expression of ~100 genes, with the vast majority being positively regulated [6]. In particular, the expression of all of the genes on the *novicida* pathogenicity island was found to be positively regulated by MglA. In addition, MglA was found to regulate the expression of virulence genes located outside of the pathogenicity island, as well as other genes not thought to play a role in virulence [6]. If MglA and SspA primarily function together to regulate gene expression, then we would predict that MglA and SspA should regulate expression of the same set of genes. To test this prediction on a genome-wide scale, we performed DNA microarray analyses to compare the global gene expression profiles of Δ*mglA* mutant cells, Δ*sspA* mutant cells, LVS wild-type cells, and cells of the control strain LVS::*FTL0951*. The results of these analyses show that in LVS, MglA and SspA regulate expression of essentially the same set of genes (Figure 5; Table 1). Specifically, we found that the expression of 30 genes changed by a factor of 3 or more when *mglA* was deleted, and the expression of 28 genes changed by a factor of 3 or more when *sspA* was deleted (Table 1). Furthermore, of the 33 genes whose expression was altered by a factor of 3 or more in either the Δ*mglA* or the Δ*sspA* mutant cells compared to wild-type cells, 25 showed at least a 3-fold change in expression in the other mutant background (Figure 5; Table 1), and all showed at least a 2-fold change in expression in the other mutant background. Because expression of five of the MglA- or SspA-regulated genes was also altered in cells of our slow growing control strain (LVS::*FTL0951*) when compared to wild-type cells, we do not necessarily know whether these particular genes are truly regulated by MglA or SspA; their expression might alter in the Δ*mglA* and Δ*sspA* mutants simply because these five genes are growth-rate regulated. The results of our microarray analyses thus show not only that MglA and SspA co-regulate expression of essentially the same set of genes in *F. tularensis,* but also that MglA and SspA exert similar effects on these target genes, as might be expected if MglA and SspA were to function together primarily as a heteromeric complex.

**Table 1:**
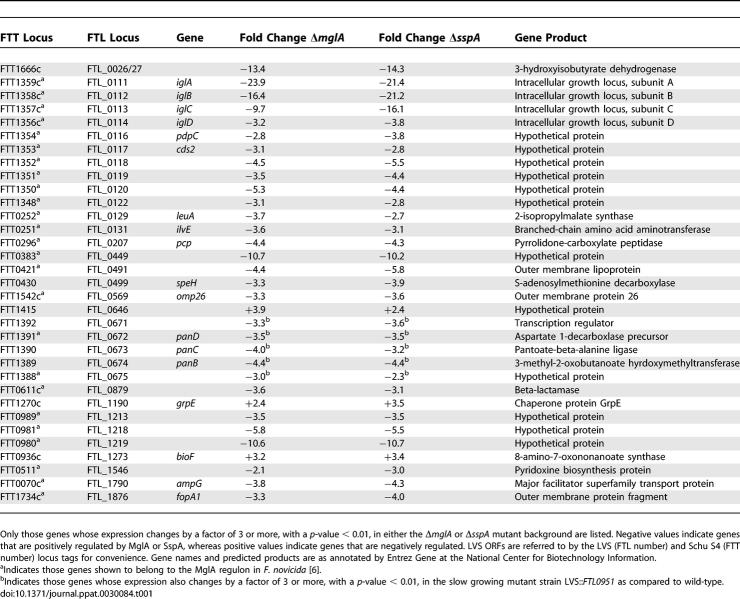
Microarray Analysis of Genes Whose Expression Changes by a Factor of 3 or More in either a Δ*mglA* or a Δ*sspA* Mutant Background Compared to Wild-Type

## Discussion

We have found that virulence gene expression is controlled by both MglA and SspA in F. tularensis. Specifically, we have shown that both MglA and SspA are RNAP-associated proteins in *F. tularensis,* and that MglA and SspA can interact with one another directly to form a heteromeric complex. Although MglA and SspA may also form homomeric complexes, we present evidence that the ability of MglA to associate with RNAP is dependent upon SspA. One implication of this finding is that members of the SspA protein family likely associate with RNAP as an oligomer, and presumably as a dimer [8,19]. Our findings suggest that although MglA homomers might form in the cell, they are unlikely to associate with RNAP. Unfortunately, we were unable to determine whether the association of SspA with RNAP is dependent upon MglA, and so we do not currently know whether SspA homomers can associate with RNAP; we were unable to construct a strain of F. tularensis containing a chromosomally TAP-tagged SspA analogous to the LVS MglA-TAP strain, presumably because integration of the *sspA*-TAP vector into the F. tularensis chromosome results in polar effects on the expression of genes downstream of *sspA* that are important for growth.

In support of the idea that MglA and SspA function together as a heteromeric complex, the results of our microarray analyses show that the MglA and SspA regulons are almost identical. In particular, we show that 30 genes belong to the MglA regulon and 28 belong to the SspA regulon, and that essentially the same set of genes are found in both (Figure 5; Table 1).

In general, the results of our microarray analyses with the Δ*mglA* mutant strain are in good agreement with those of Brotcke and colleagues, who analyzed the effects of an *mglA* point mutation on gene expression in F. novicida [6] (see Table 1). Note that when defining the MglA regulon, we listed those genes whose expression changed by a factor of 3 or more when *mglA* was deleted. If we had defined the regulon by listing those genes whose expression changed by a factor of 2 or more, then the MglA regulon would be composed of more than 100 genes (Table S1).

**Figure 6:**
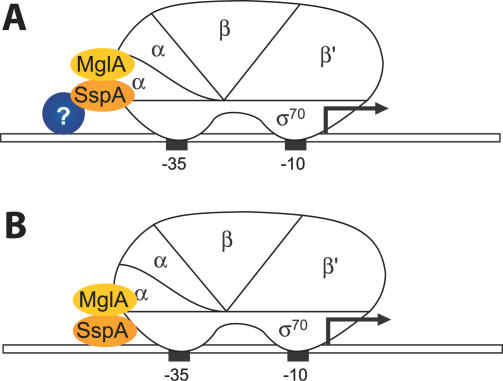
Models for How the MglA–SspA Complex Positively Controls Virulence Gene Expression in F. tularensis (A) Contact between a DNA-bound transcription activator and the RNAP-associated MglA–SspA complex activates transcription from a virulence gene promoter. The arrow indicates the transcription start site. (B) Contact between promoter DNA and the RNAP-associated MglA–SspA complex activates transcription from a virulence gene promoter. In these models, the virulence gene promoters are depicted as being recognized by RNAP holoenzyme containing σ^70^. The RNAP that co-purifies with MglA-TAP appears to contain σ^70^ in stoichiometric amounts (Figure 1B), suggesting that the promoters of MglA/SspA-dependent genes may be σ^70^-dependent.

Although the molecular details of how MglA exerts its effects on virulence gene expression (or on the expression of any other target gene) are not yet known, we have presented evidence that its association with RNAP plays an important, if not essential, role. For example, because expression of the *iglA, iglC,* and *pdpA* virulence genes is downregulated in a Δ*sspA* mutant, and because MglA is present but is not associated with RNAP in a Δ*sspA* mutant, we disfavor any model whereby MglA upregulates these genes without being associated with RNAP. Accordingly, we suggest two possible models for how MglA, together with SspA, positively controls virulence gene expression in F. tularensis based on association of the MglA–SspA complex with RNAP. The first model specifies that a DNA-bound transcription activator(s) contacts the RNAP-associated MglA–SspA complex directly to stimulate transcription initiation at target promoters. Put another way, we hypothesize that the MglA–SspA complex effectively becomes a “subunit” of RNAP that serves as a target for DNA-bound transcription activators that regulate the expression of virulence genes in F. tularensis (see Figure 6A). Precedent for such a model comes from studies of bacteriophage P1 late gene expression. In particular, the P1 late promoter activator (Lpa or gp10) is a phage-encoded, sequence-specific DNA binding protein that is thought to activate P1 late gene expression through a direct interaction with E. coli SspA [11]. In this particular situation, E. coli SspA evidently functions as a co-activator of P1 late gene expression by making simultaneous contact with RNAP and DNA-bound Lpa [11]. In F. tularensis there may be Lpa-like proteins (at least functionally) that bind the MglA–SspA complex and are required for the co-activation of MglA- and SspA-dependent virulence gene expression (see Figure 6A). Such MglA- and/or SspA-binding proteins, if they do indeed exist, would be predicted to be DNA-binding proteins that bind the promoter regions of MglA- and SspA-dependent virulence genes.

The second model specifies that the RNAP-associated MglA–SspA complex interacts directly with the promoter DNA of MglA- and SspA-dependent virulence genes to activate transcription initiation from the cognate promoters (Figure 6B). However, there is no evidence that any SspA homolog is capable of binding directly to DNA; in particular, E. coli SspA does not appear to bind P1 late promoter DNA [11]. Other models can also be envisioned. These include the MglA–SspA complex modifying the promoter-recognition properties of the associated RNAP holoenzyme without interacting directly with the DNA, or modifying the elongation properties of the associated RNAP. We note that the latter model would be unlikely if the MglA–SspA complex, like SspA in E. coli [8], could interact only with the RNAP holoenzyme and not with the core enzyme and therefore was not a component of mature elongation complexes.

The models discussed above and depicted in Figure 6 are meant only to explain how MglA and SspA positively regulate the expression of target genes. However, MglA and SspA also appear to negatively regulate the expression of at least some target genes (Table 1; [6]). Whether those genes whose expression is negatively controlled by MglA and SspA are controlled directly, or indirectly, remains to be determined.

F. tularensis is unique in that it contains two genes, designated *rpoA1* and *rpoA2,* encoding different α subunits of RNAP. This situation is without precedent; in all other bacterial genomes sequenced to date there is only one *rpoA* gene. The two α subunits are 32% identical and 55% similar to one another at the primary amino acid level and differ from one another in regions that may be important for dimer formation, promoter recognition, and interaction with DNA-bound transcription activators. We have shown that both α1 and α2 are components of RNAP in F. tularensis. Because α is a dimer in the RNAP complex, there could be as many as four different species of RNAP core enzyme in *F. tularensis;* one composed of α1 homodimers, one composed of α2 homodimers, and two composed of α1α2 heterodimers (that differ from one another with respect to which α protomer interacts with the β subunit of RNAP and which α protomer interacts with β′). However, a subset of the predicted dimerization determinants [23] present in the N-terminal domains of α1 and α2 differ from one another, raising the possibility that α1 and α2 might exclusively form either homodimers or heterodimers. The experiments we have performed do not allow us to say anything more than that both α1 and α2 are subunits of RNAP in F. tularensis. Because α participates in promoter recognition through direct sequence-specific protein–DNA interactions [16,24], and because α is a common target for transcription activators [25], the presence of RNAP species containing distinct α subunits might significantly affect the control of gene expression in F. tularensis.

The F. tularensis RNAP complex is also unusual in that it is associated with two different members of the SspA protein family (MglA and SspA). Other bacterial pathogens where SspA homologs play roles in virulence gene control, such as Neisseria gonorrhoeae [12], Yersinia enterocolitica [13], and Vibrio cholerae [14], appear to encode only one SspA homolog. Whether the use of two SspA homologs allows for the integration of multiple environmental signals, or is related to the fact that the F. tularensis RNAP contains two distinct α subunits, or both, remains to be determined.

## Materials and Methods

### Plasmids, strains, and growth conditions.

F. tularensis subspecies *holarctica* strain LVS was provided by Karen Elkins (Food and Drug Administration, Rockville, Maryland, United States). LVS was grown at 37 °C in modified Mueller Hinton (MH) broth (Difco, http://www.bd.com) supplemented with glucose (0.1%), ferric pyrophosphate (0.025%), and Isovitalex (2%), or on cysteine heart agar (Difco) medium supplemented with 1% hemoglobin solution (VWR, http://www.vwrsp.com); when appropriate, kanamycin was used for selection at 5 μg/ml. E. coli strains XL1-blue (Stratagene, http://www.stratagene.com) and DH5αF′IQ (Invitrogen, http://www.invitrogen.com) were used as recipients for all plasmid constructions. E. coli strain KDZif1ΔZ was used as the reporter strain for the bacterial two-hybrid experiments. KDZif1ΔZ harbors an F′ episome containing the *lac* promoter derivative p*lac*Zif1–61 driving expression of a linked *lacZ* reporter gene and has been described previously [22]. E. coli strain BL21-CodonPlus (DE3)-RP (Stratagene) was used for the overproduction of recombinant proteins. The deletion constructs for *mglA* and *sspA* were generated by amplifying flanking regions by the PCR and then splicing the flanking regions together by overlap-extension PCR; deletions were in-frame and contained the first three codons and last three codons of the open reading frame (ORF) separated by the 9-bp linking sequence 5′–GCGGCCGCC–3′. The resulting PCR products were cloned into plasmid pEX18km (provided by Shite Sebastian and Simon Dillon, Harvard Medical School, Boston, Massachusetts, United States), yielding pEX-Δ*mglA* and pEX-Δ*sspA*. Plasmid pEX18km contains a ColE1 origin of replication (which is not functional in LVS), the Tn903 kanamycin resistance gene (Epicentre, http://www.epibio.com) expressed from the LVS *groEL* promoter, and a *sacB* gene. Because the *sacB* gene on pEX18km is insufficient to mediate efficient sucrose counterselection in LVS, a second copy of the *sacB* gene was subcloned from plasmid pPV [26] on a PstI fragment into plasmids pEX-Δ*mglA* and pEX-Δ*sspA* that had been digested with PstI, creating plasmids pEX2-Δ*mglA* and pEX2-Δ*sspA,* respectively. Plasmids pEX2-Δ*mglA* and pEX2-Δ*sspA* were then used to create strains LVS Δ*mglA* and LVS Δ*sspA* by allelic exchange (see [26]). Deletions were confirmed by the PCR and by Southern blotting.

Strain LVS::*FTL0951* contains a mariner transposon conferring resistance to kanamycin integrated into the LVS chromosome at the FTL_0951 locus; the presence of a single transposon in this strain was confirmed by Southern blotting, and the location of the transposon was determined by sequencing of the chromosomal DNA.

*Complementation vectors:* Plasmid pF-MglA was used for complementation of the LVS Δ*mglA* mutant strain and was constructed by inserting full-length *mglA* in place of *gfp* in the plasmid pFNLTP6 *gro*-*gfp* [27]. Plasmid pF was used as a control for complementation experiments and was constructed by deleting the *gfp* gene from plasmid pFNLTP6 *gro*-*gfp* [27]. Plasmid pF2-SspA was used for complementation of the LVS Δ*sspA* mutant strain and was constructed in two steps. First, full-length *sspA* was inserted into pFNLTP6 *gro*-*gfp* [27] in place of the *gfp* gene creating plasmid pF-SspA. Second, to compensate for the apparent deleterious effect of overexpressing *sspA* in *F. tularensis,* the full-length LVS *groEL* promoter in pF-SspA was replaced with a more minimal *groEL* promoter lacking the putative UP-element. As a control, plasmid pF2 was constructed by replacing the full-length LVS *groEL* promoter in pF with the same minimal *groEL* promoter used in plasmid pF2-SspA.

*Plasmids and strains for TAP-tag experiments:* Plasmids for generating TAP-tag strains were constructed by inserting the last ~400 bp of the ORF into the vector pEXTAP (this work; Figure 1A), which contains the TAP-tag sequence from plasmid pP30ΔYTAP [22] cloned into the multiple cloning site of pEX18km. Specifically, plasmid pEX-MglA-TAP was made by cloning an ~400-bp fragment of DNA corresponding to a 3′ portion of the *mglA* gene into pEXTAP such that the *mglA* portion was in-frame with the DNA specifying the TAP-tag. Strain LVS MglA-TAP was constructed by electroporating pEX-MglA-TAP into LVS (essentially as described in [27]) and selected on cysteine heart agar medium containing hemoglobin (1%) and kanamycin (5 μg/ml). Because pEX-MglA-TAP cannot replicate in *F. tularensis,* only those cells in which the plasmid has integrated into the chromosome can grow on media containing kanamycin. Strains LVS SucD-TAP and LVS β′-TAP were made in a similar fashion using plasmids pEX-SucD-TAP and pEX-RpoC-TAP, respectively. Strain LVS Δ*sspA* MglA-TAP was made using plasmid pEX-MglA-TAP together with strain LVS Δ*sspA*. For all strains, insertion of the TAP integration vector at the correct chromosomal location was confirmed by the PCR, and production of the corresponding TAP-tagged protein was confirmed by Western blotting with PAP [28], which binds the ProtA moieties of the TAP-tag.

*Plasmids for bacterial two-hybrid assays:* Plasmid pBRGPω directs the synthesis of the Gal11P-ω fusion protein and has been described before [22]. Plasmid pBRGPω can be used to create fusions to the N-terminus of the ω subunit of E. coli RNAP; proteins are fused to ω via a small linker composed of three alanine residues specified in part by a NotI restriction site. Plasmid pBRGPω confers resistance to carbenicillin, harbors a ColE1 origin of replication, and carries an IPTG-inducible *lac*UV5 promoter that drives expression of the ω fusion protein [22]. Plasmids pBRMglA-ω and pBRSspA-ω direct the synthesis of either full-length MglA or full-length LVS SspA fused to residues 2–90 of E. coli ω. The pBR-ω fusion plasmids were made by cloning the appropriate NdeI-NotI–digested PCR products into NdeI-NotI–digested pBRGPω. Each of the respective ω fusion proteins is therefore under the control of an IPTG-inducible *lac*UV5 promoter.

Plasmid pACTR-AP-Zif directs the synthesis of Zif, the zinc finger DNA-binding domain of the murine Zif268 protein, and has been described previously [22]. This plasmid can be used to create fusions to the N-terminus of Zif; proteins are fused to Zif via a nine–amino acid linker (Ala-Ala-Ala-Pro-Arg-Val-Arg-Thr-Gly) specified in part by a NotI restriction site. Plasmid pACTR-AP-Zif confers resistance to tetracycline and harbors a p15A origin of replication. Plasmids pACTR-MglA-Zif and pACTR-SspA-Zif direct the synthesis of either full-length MglA (residues 1–205) or full-length LVS SspA (residues 1–210) fused to Zif. The pACTR-Zif fusion plasmids were made by cloning the appropriate NdeI-NotI–digested PCR products into NdeI-NotI–digested pACTR-AP-Zif. Each of the respective Zif fusion proteins is therefore under the control of an IPTG-inducible *lac*UV5 promoter.

### TAP.

Cells were grown at 37 °C with aeration in 200 ml of MH broth supplemented with glucose (0.1%), ferric pyrophosphate (0.025%), and Isovitalex (2%). Cells were grown to an OD_600_ of ~0.4 and harvested by centrifugation at 4 °C. TAP was then performed essentially as described earlier [18].

### Bacterial two-hybrid assays.

Cells were grown with aeration at 37 °C in LB supplemented with kanamycin (50 μg/ml), carbenicillin (100 μg/ml), tetracycline (10 μg/ml), and IPTG at the concentration indicated. Cells were permeabilized with SDS-CHCl_3_ and assayed for β-galactosidase activity as described previously [29]. Assays were performed at least three times in duplicate on separate occasions. Representative data sets are shown. Values are averages based on one experiment; duplicate measurements differed by less than 10%.

### Co-purification assays.

Plasmid pETDuetMglA-S directs the synthesis of MglA with a C-terminal S-tag (MglA-S) and confers resistance to carbenicillin, while plasmid pRSFDuetSspA-His6 directs the synthesis of SspA with a C-terminal hexa-histidine tag (SspA-His6) and confers resistance to kanamycin. Plasmid pRSFDuetSspA directs the synthesis of wild-type SspA and confers resistance to kanamycin. Plasmid pETDuetMglA-S was introduced into E. coli strain BL21-CodonPlus(DE3)-RP (Stratagene) along with pRSFDuetSspA-His6 or pRSFDuetSspA. Single colonies were used to inoculate Overnight Express Instant TB medium (Novagen, http://www.emdbiosciences.com/html/NVG/home.html) supplemented with kanamycin (50 μg/ml), carbenicillin (100 μg/ml), and chloramphenicol (25 μg/ml), and cultures were grown overnight at 30 °C with aeration to allow for induction. Cell pellets from 50 ml of overnight culture (OD_600_ of ~3.0) were resuspended in 1 ml of binding buffer (10 mM imidazole; 10 mM Tris-HCl [pH 8.0]; 300 mM NaCl; 10% glycerol; and 1X EDTA-free protease inhibitor cocktail [Roche, http://www.roche.com]). Each 1-ml suspension was separated into 500 μl aliquots, and 100 μl of lysozyme (10 mg/ml) was added to each aliquot. Cells were then incubated on ice for 1 h and lysed by sonication, and cell debris was removed from each lysate by centrifugation. Lysates were then added to TALON polyhistidine-tag purification resin (Clontech, http://www.clontech.com), and following washing with binding buffer, proteins specifically bound to the resin were eluted in buffer containing 250 mM imidazole, 10 mM Tris-HCl (pH 8.0), 300 mM NaCl, 10% glycerol, and 1X EDTA-free protease inhibitor cocktail.

### Immunoblots.

Cell lysates and eluted proteins were separated by SDS-PAGE on 4%–12% Bis-Tris NuPAGE gels in MOPS running buffer (Invitrogen) and transferred to nitrocellulose using the iBlot dry blotting system (Invitrogen). Membranes were blocked with 25 ml of SuperBlock Blocking Buffer (Pierce, http://www.piercenet.com) in TBS with 250 μl Surfact-Amps 20 (Pierce). Membranes were then probed with monoclonal antibodies against the S-Tag (Novagen), the His-Tag (Novagen), or the α subunit of E. coli RNAP (NeoClone, http://www.neoclone.com). Proteins were detected using goat polyclonal anti-mouse IgG conjugated with horseradish peroxidase (Pierce), and visualized using SuperSignal West Pico Chemiluminescent Substrate (Pierce).

### RNA isolation and quantitative RT-PCR.

Cells were grown with aeration at 37 °C in MH broth supplemented with glucose (0.1%), ferric pyrophosphate (0.025%), and Isovitalex (2%). RNA isolation and cDNA synthesis were essentially as described previously for Pseudomonas aeruginosa [30]. Transcript quantities for *iglA, iglC,* and *pdpA* were determined relative to the amount of *tul4* transcript by RT-PCR with the iTaq SYBR Green kit (Bio-Rad, http://www.bio-rad.com) and an ABI Prism 7000 Sequence Detection System (Applied Biosystems, http://www.appliedbiosystems.com); the REST 2005 software package was used for statistical analysis of the data [31].

### Array design and construction.

ORFs for LVS were predicted using GLIMMER [32] and annotated by comparison with the annotated Schu S4 genome sequence using BLAST [15]. ORFs were manually annotated guided by BLAST results but with appropriate alterations. For example, ORFs containing sequencing errors were identified as being those where adjacent genes were fully contained within predicted Schu S4 ORFs.

The DNA microarray consisted of sequence fragments constructed by PCR. Primers for PCR were designed using PRIMER3 ([33]; http://primer3.sourceforge.net) software using the genome annotation. The 1,932 primer sets produced amplicons of 42 bp to 400 bp in length centrally located within each gene. They correspond to sequences for 1,678 LVS ORFs with Schu S4 homologs (including the 15 duplicated ORFs in the pathogenicity island), 24 GLIMMER-predicted ORFs with no homology to Schu S4, and 230 intergenic regions. Greater than 95% of LVS non-transposase genes were represented on the microarray. The primer sets contained 5′ extensions to each forward and reverse primer, GGCATCTAGAGCAC and CCGCACTAGTCCTC, respectively.

All amplifications were performed using a standard protocol. In the first round of amplification we used 20-μl PCR reactions containing 1 ng of LVS genomic DNA as template, 20 pmol of unique primers, and 5 units of Taq polymerase (Takara, http://www.takarabiousa.com). These reactions were then diluted 1:150. A second round contained 1 μl of the diluted first reaction as template, 20 pmol of universal primers containing 5′ amino modification including a 3-carbon linker (GAACCGATAGGCATCTAGAGCAC and GAAATCCACCGCACTAGTCCTC), and 5 units of Taq polymerase in a total volume of 100 μl. Aliquots (5 μl) of each first and second round reaction were run on 2% agarose gels to confirm the presence of appropriately sized products. The PCR products were purified using 96-well QiaQuick Multiwell PCR Purification plates (Qiagen, http://www.qiagen.com), eluted in deionized water, and transferred to 384-well plates. Purified products were air-dried in a biosafety cabinet at room temperature and resuspended in 50 mM sodium phosphate (pH 8.5). Each PCR amplicon was printed in duplicate onto CodeLink Activated Slides (Amersham, http://www.amersham.com) using a Qarrayer (Genetix, http://www.genetix.com). The microarrays were then post-processed according to the slide manufacturer's instructions.

### Microarray experiments.

Strains were grown to mid-log in supplemented MH medium and RNA was isolated from 15 ml of culture using Qiagen RNeasy Midi columns. Samples were DNase-treated twice: on the columns using a Qiagen RNase-Free DNase Set, and after purification using RQ1 DNase (Promega, http://www.promega.com). RNA was purified from three separate cultures for each strain. cDNA was synthesized using ~10 μg RNA, 750 ng NSNSNSNSNS random primers (where N refers to any base and S refers to G or C), and Superscript II Reverse Transcriptase (Invitrogen). Amino acyl-dUTP (aa-dUTP) was incorporated into the cDNA by including a 2:1 ratio of aa-dUTP to dTTP in the reverse transcription reaction. RNA was removed by increasing the pH with NaOH, and then unincorporated aa-dUTP and free amines were removed by purifying the cDNA with Qiagen MinElute PCR Purification columns. Eluted cDNA was dried to completion in a speed-vac and then labeled with dye; in a 9-μl volume, wild-type LVS cDNA was labeled with Cy5 dye (Amersham; Cy5 mono-Reactive Dye Pack) in 0.1 M sodium carbonate (pH 9.3), and cDNA from LVS Δ*mglA,* LVS Δ*sspA,* and LVS::*FTL0951* were similarly labeled with Cy3 dye (Amersham; Cy3 mono-Reactive Dye Pack). After coupling, 35 μl of 100 mM NaOAc (pH 5.2) was added to each reaction and free dye was removed using Qiagen MinElute PCR purification columns. cDNA equivalent to ~600 pmol Cy5 dye was mixed with cDNA equivalent to ~600 pmol Cy3 dye from either LVS Δ*mglA,* LVS Δ*sspA,* or LVS::*FTL0951* and then dried to completion in a speed-vac. The labeled probes were resuspended in hybridization buffer (5X SSC, 50% formamide, 0.1% SDS, 0.2 mg/ml tRNA), and applied to arrays using a Tecan HS400 Hybridization Station. Arrays were scanned using a GenePix 4000B microarray scanner (Molecular Devices, http://www.moleculardevices.com) and data were analyzed with GeneSpring GX.

## Supporting Information

Figure S1 Effect of Growth Phase on Abundance of the *mglA* and *sspA* Transcripts in F. tularensis
Quantitative RT-PCR analysis of *mglA* and *sspA* transcript levels in cells of the LVS wild-type strain at different phases of growth. The mid-logarthmic (mid-log), late-log, and stationary phase cultures had OD_600_'s of 0.4, 1.0, and 2.6, respectively. Transcripts were normalized to *tul4* whose expression remains relatively constant throughout the growth curve; compared to cells in mid-log, cells in late-log and stationary phase had 1.03 times (standard error [SE] = 0.21) and 1.64 times (SE = 0.12) the number of *tul4* transcripts, respectively, as determined by quantitative RT-PCR.
(136 KB PDF)

Figure S2 Complementation of the Effects of the Δ*mglA* and Δ*sspA* Mutations on Virulence Gene Expression in F. tularensis
Quantitative RT-PCR analysis of *iglA, iglC,* and *pdpA* transcript levels in wild-type, Δ*mglA,* and Δ*sspA* mutant cells containing the indicated plasmids. Transcripts were normalized to *tul4* whose expression is not influenced by MglA or SspA. Plasmids pF-MglA and pF2-SspA direct the synthesis of MglA and SspA, respectively. Plasmids pF and pF2 served as empty vector controls for plasmids pF-MglA and pF2-SspA, respectively.
(548 KB PDF)

Table S1 Microarray Analysis of Genes Whose Expression Changes by a Factor of 2 or More in either a Δ*mglA* or a Δ*sspA* Mutant Background Compared to Wild-Type (247 KB DOC)

### Accession Numbers

Proteins discussed in this study, followed by their corresponding locus tag and Entrez Protein (http://www.ncbi.nlm.nih.gov/entrez/query.fcgi?db=Protein) accession numbers are as follows: α1 (FTL_0261; YP_513056.1), α2 (FTL_0616; YP_513375.1), MglA (FTL_1185; YP_513868), and SspA (FTL_1606; YP_514245.1). The Entrez Genome (http://www.ncbi.nlm.nih.gov/entrez/query.fcgi?db=Genome) accession number for the nucleotide sequence of the F. tularensis LVS genome is NC_007880.1.

## Abbreviations

LVS: live vaccine strain
MglA: macrophage growth locus A
MS/MS: tandem mass spectrometry
ORF: open reading frame
RNAP: RNA polymerase
RT-PCR: reverse transcriptase PCR
SE: standard error
SspA: stringent starvation protein A
TAP: tandem affinity purification
