# Supplementary material for: Twin RNA Polymerase–Associated Proteins Control Virulence Gene Expression in Francisella tularensis
Source: PLoS Pathog. 2007 Jun 15;3(6):e84. doi: 10.1371/journal.ppat.0030084 (PMC1891329; doi:10.1371/journal.ppat.0030084)
Supplement: Supplementary file 1 [file ppat.0030084.sg001.pdf]

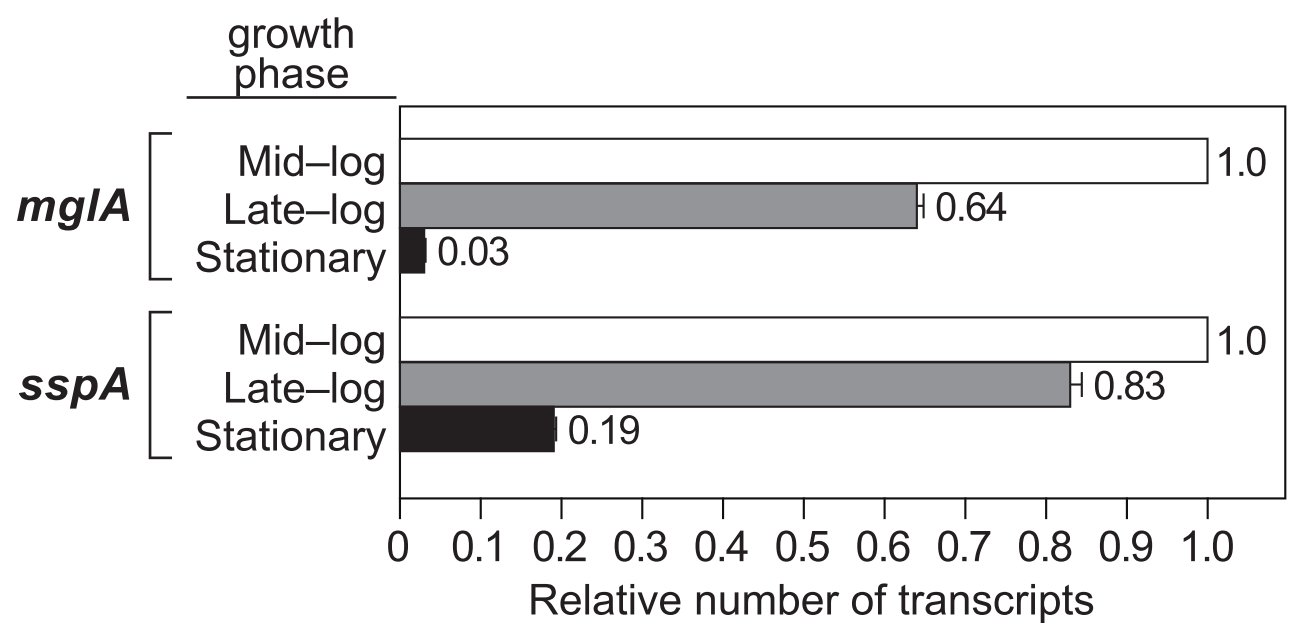

growth phase
mglA
Mid–log
Late–log
Stationary
1.0
0.64
0.03
sspA
Mid–log
Late–log
Stationary
1.0
0.83
0.19
0 0.1 0.2 0.3 0.4 0.5 0.6 0.7 0.8 0.9 1.0
Relative number of transcripts
