# Supplementary material for: Twin RNA Polymerase–Associated Proteins Control Virulence Gene Expression in Francisella tularensis
Source: PLoS Pathog. 2007 Jun 15;3(6):e84. doi: 10.1371/journal.ppat.0030084 (PMC1891329; doi:10.1371/journal.ppat.0030084)
Supplement: Supplementary file 2 [file ppat.0030084.sg002.pdf]

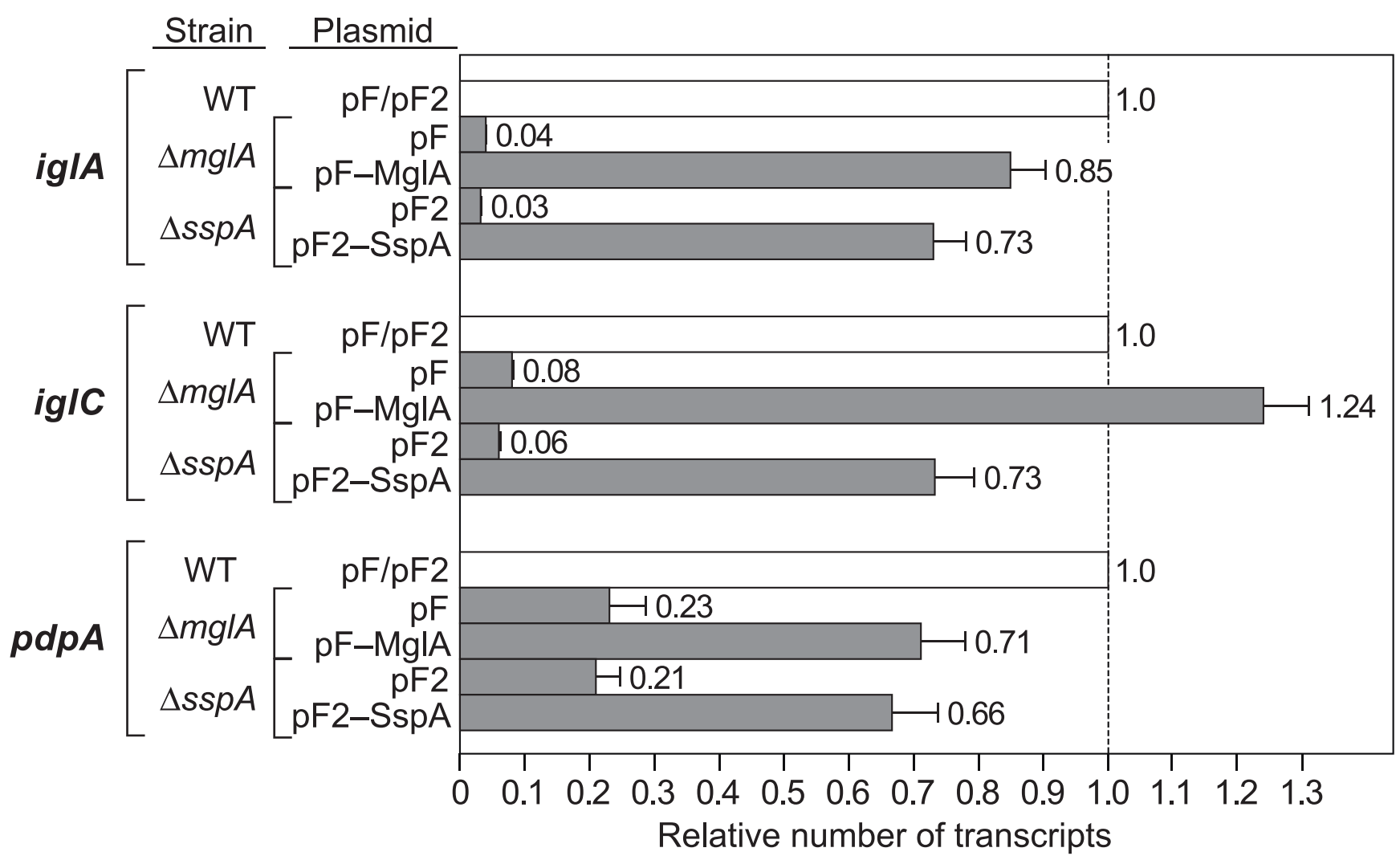
Strain
Plasmid
iglA
WT
pF/pF2
1.0
ΔmglA
pF
0.04
pF–MglA
0.85
ΔsspA
pF2
0.03
pF2–SspA
0.73
iglC
WT
pF/pF2
1.0
ΔmglA
pF
0.08
pF–MglA
1.24
ΔsspA
pF2
0.06
pF2–SspA
0.73
pdpA
WT
pF/pF2
1.0
ΔmglA
pF
0.23
pF–MglA
0.71
ΔsspA
pF2
0.21
pF2–SspA
0.66
0 0.1 0.2 0.3 0.4 0.5 0.6 0.7 0.8 0.9 1.0 1.1 1.2 1.3
Relative number of transcripts
