# Supplementary material for: Twin RNA Polymerase–Associated Proteins Control Virulence Gene Expression in Francisella tularensis
Source: PLoS Pathog. 2007 Jun 15;3(6):e84. doi: 10.1371/journal.ppat.0030084 (PMC1891329; doi:10.1371/journal.ppat.0030084)
Supplement: Supplementary file 3 [file ppat.0030084.st001.doc]

Table S1. Microarray analysis of genes whose expression changes by a factor of 2 or more in either a Δ*mglA* or a Δ*sspA* mutant background compared to wild-type.

| FTT locus | FTL locus | Gene | Δ*mglA* Fold change | Δ*sspA* Fold change | Gene product |
| --- | --- | --- | --- | --- | --- |
| FTT1666c | FTL0026/FTL0027 |  | -13.4 | -14.3 | 3-hydroxyisobutyrate dehydrogenase |
| FTT1653 | FTL0039 |  | +1.7 | +2.1 | Hypothetical membrane protein |
| FTT1690 | FTL0056 |  | +2.5 | +2.0 | NADH dehydrogenase |
| FTT1684 | FTL0062 |  | +2.4 | +1.6 | Transcription regulator |
| FTT1771 | FTL0097 |  | -2.5 | -4.2 | Hypothetical protein |
| FTT1773c | FTL0099 | *trpB* | -2.6 | -2.0 | Tryptophan synthase, subunit beta |
| FTT1359c | FTL0111 | *iglA* | -23.9 | -21.4 | Intracellular growth locus, subunit A |
| FTT1358c | FTL0112 | *iglB* | -16.4 | -21.2 | Intracellular growth locus, subunit B |
| FTT1357c | FTL0113 | *iglC* | -9.7 | -16.1 | Intracellular growth locus, subunit C |
| FTT1356c | FTL0114 | *iglD* | -3.2 | -3.8 | Intracellular growth locus, subunit D |
| FTT1354 | FTL0116 | *pdpC* | -2.8 | -3.8 | Hypothetical protein |
| FTT1353 | FTL0117 | *cds2* | -3.1 | -2.8 | Hypothetical protein |
| FTT1352 | FTL0118 |  | -4.5 | -5.5 | Hypothetical protein |
| FTT1351 | FTL0119 |  | -3.5 | -4.4 | Hypothetical protein |
| FTT1350 | FTL0120 |  | -5.3 | -4.4 | Hypothetical protein |
| FTT1348 | FTL0122 |  | -3.1 | -2.8 | Hypothetical protein |
| FTT0252 | FTL0129 | *leuA* | -3.7 | -2.7 | 2-isopropylmalate synthase |
| FTT0251 | FTL0131 | *ilvE* | -3.6 | -3.1 | Branched-chain amino acid aminotransferase |
| FTT0218c | FTL0155 |  | -2.0 | -2.8 | Cytochrome b561 family protein, pseudogene |
| FTT0233c | FTL0178 | *yidC* | +2.4 | +2.0 | Inner-membrane protein |
| FTT0296 | FTL0207 | *pcp* | -4.4 | -4.3 | Pyrrolidone-carboxylate peptidase |
| FTT0298 | FTL0209 | *holC* | -2.7 | -3.3 | DNA polymerase III, CHI subunit |
| FTT0311c | FTL0222 |  | -1.9 | -2.1 | Hypothetical protein |
| FTT0356 | FTL0267 | *htpG* | +1.9 | +2.2 | Chaperone heat shock protein HtpG |
| FTT0380c | FTL0269 | *gdh* | -1.9 | -2.1 | Glutamate dehydrogenase |
| FTT1498c | FTL0295 | *accA* | -3.0 | -2.2 | Acetyl-coenzyme A carboxylase carboxyl transferase, subunit alpha |
| FTT1487 | FTL0307 | *coaE* | -2.3 | -1.9 | Dephospho-CoA kinase |
| FTT0831c | FTL0325 |  | -2.3 | -1.9 | OmpA family protein |
| FTT0852 | FTL0348 |  | -6.2 | -7.0 | Conserved hypothetical protein, pseudogene |
| FTT0865 | FTL0365 |  | +2.0 | +1.4 | Hypothetical protein |
| FTT0875c | FTL0376 |  | +2.0 | +2.4 | Hypothetical protein |
| FTT0883 | FTL0385/0386 |  | -1.7 | -2.1 | Alcohol dehydrogenase, pseudogene/Glutathione dependent formaldehyde dehydrogenase |
| FTT0905 | FTL0425 |  | -2.4 | -2.3 | Type IV pili glycosylation protein |
| FTT0383 | FTL0449 |  | -10.7 | -10.2 | Hypothetical protein |
| FTT0384c | FTL0450 | *psd* | +2.1 | +2.4 | Phosphatidylserine decarboxylase proenzyme |
| FTT0403 | FTL0473 | *def1* | -2.2 | -2.4 | Peptide deformylase |
| FTT0407 | FTL0477 | *gcvT* | -2.1 | -2.4 | Aminomethyltransferase |
| FTT0410 | FTL0480 | *gcvP2* | -2.0 | -1.9 | Glycine dehydrogenase subunit 2 |
| FTT0415 | FTL0485 | *glgC* | +2.0 | +2.4 | Glucose-1-phosphate adenylyltransferase |
| FTT0416 | FTL0486 | *glgA* | +2.2 | +2.1 | Glycogen synthase |
| FTT0421 | FTL0491 |  | -4.4 | -5.8 | Outer membrane lipoprotein |
| FTT0430 | FTL0499 | *speH* | -3.3 | -3.9 | S-adenosylmethionine decarboxylase |
| FTT0431 | FTL0500 | *speE* | -4.0 | -2.5 | Spermidine synthase |
| FTT1579c | FTL0528 |  | +2.9 | +3.2 | Type III restriction enzyme |
| FTT1578c | FTL0529 | *Ung* | +3.3 | +1.9 | Uracil-DNA glycosylase |
| FTT1564 | FTL0544 |  | +2.1 | +2.2 | Hypothetical protein |
| FTT1542c | FTL0569 | *omp26* | -3.3 | -3.6 | Outer membrane protein 26 |
| FTT1539c | FTL0572 |  | -2.3 | -2.3 | Hypothetical protein |
| FTT1529 | FTL0585 | *fadE* | -2.0 | -2.3 | Acyl-CoA dehydrogenase |
| FTT1450c | FTL0606 | *wbtM* | +2.1 | +1.2 | dTDP-D-glucose 4,6-dehydratase |
| FTT1441 | FTL0617 |  | -2.5 | -2.0 | Hypothetical protein |
| FTT1428c | FTL0630 |  | +2.1 | +1.8 | Acetyltransferase |
| FTT1415 | FTL0646 |  | +3.9 | +2.4 | Hypothetical protein |
| FTT1400c | FTL0663 |  | -2.4 | -1.5 | Hypothetical protein |
| FTT1392 | FTL0671 |  | -3.3 | -3.6 | Transcription regulator |
| FTT1391 | FTL0672 | *panD* | -3.5 | -3.5 | Aspartate 1-decarboxlase precursor |
| FTT1390 | FTL0673 | *panC* | -4.0 | -3.2 | Pantoate-beta-alanine ligase |
| FTT1389 | FTL0674 | *panB* | -4.4 | -4.4 | 3-methyl-2-oxobutanoate hyrdoxymethyltransferase |
| FTT1388 | FTL0675 |  | -3.0 | -2.3 | Hypothetical protein |
| FTT1258 | FTL0686 |  | +2.1 | +1.4 | Outer membrane efflux protein |
| FTT1201c | FTL0743 |  | -2.4 | -2.3 | Oxidoreductase, short-chain dehydrogenase family protein |
| FTT1180 | FTL0767 |  | -2.3 | -2.3 | Hypothetical protein |
| FTT1168c | FTL0786 | *nagA* | -2.3 | -1.9 | N-acetylglucosamine-6-phosphate deacetylase |
| FTT1161 | FTL0795 | *adk* | -2.1 | -1.7 | Adenylate kinase |
| FTT1149c | FTL0806 |  | +2.0 | +1.3 | Amino acid transporter family protein |
| FTT0595c | FTL0859/FTL0860 | *rubA* | -2.1 | -2.5 | Rubredoxin/Hypothetical protein |
| FTT0611c | FTL0879 |  | -3.6 | -3.1 | Beta-lactamase |
| FTT0613c | FTL0881 |  | -2.7 | -3.1 | Hypothetical protein |
| FTT0665c | FTL0939 |  | +1.6 | +2.3 | Aldolase/adducin class II family protein |
| FTT0668 | FTL0942 |  | -2.2 | -2.3 | Nicotinamide mononucleotide transport (NMT) family protein |
| FTT0556c | FTL1014 | *oxyR* | -2.1 | -1.8 | Oxidative stress transcription regulator |
| FTT1035c | FTL1050 | *rpoD* | +2.7 | +2.4 | RNA polymerase sigma-70 factor |
| FTT1103 | FTL1090 |  | -2.3 | -2.2 | Conserved hypothetical lipoprotein |
| FTT1098c | FTL1103 |  | +2.3 | +2.2 | DNA helicase |
| FTT1095c | FTL1107 |  | +1.8 | +2.2 | Hypothetical protein |
| FTT1379c | FTL1135/FTL1136 |  | +2.2 | +1.8 | Conserved Hypothetical protein, pseudogene/Conserved Hypothetical protein, pseudogene |
| FTT1378 | FTL1136 |  | +2.7 | +2.2 | Hypothetical protein |
| FTT1373 | FTL1141 | *fabH* | +2.2 | +2.4 | 3-oxoacyl-[acyl carrier protein] synthase III |
| FTT1368c | FTL1146 | *gapA* | -2.0 | -2.4 | Glyceraldehyde-3-phosphate dehydrogenase |
| FTT1367c | FTL1147 | *pgk* | -2.8 | -2.1 | Phosphogylcerate kinase |
| FTT1365c | FTL1149 | *fbaB* | -2.3 | -2.4 | Fructose-bisphosphate aldolase |
| FTT1278c | FTL1182 | *rnhB* | +2.1 | +1.7 | Ribonuclease H2 |
| FTT1270c | FTL1190 | *grpE* | +2.4 | +3.5 | Chaperone protein GrpE |
| FTT0991 | FTL1211 |  | -2.3 | -1.6 | Hypothetical lipoprotein |
| FTT0989 | FTL1213 |  | -3.5 | -3.5 | Hypothetical protein |
| FTT0981 | FTL1218 |  | -5.8 | -5.5 | Hypothetical protein |
| FTT0980 | FTL1219 |  | -10.6 | -10.7 | Hypothetical protein |
| FTT0976 | FTL1224 | *trxA2* | -2.6 | -2.1 | Thioredoxin 1 |
| FTT0975 | FTL1225 |  | -2.3 | -1.9 | Hypothetical protein |
| FTT0971 | FTL1230 |  | +1.9 | +2.0 | ABC transporter |
| FTT0953c | FTL1251 |  | -2.7 | -2.1 | Proton-dependent oligopeptide transport (POT) family protein |
| FTT0937c | FTL1272 | *bioB* | +2.1 | +2.7 | Biotin synthase |
| FTT0936c | FTL1273 | *bioF* | +3.2 | +3.4 | 8-amino-7-oxononanoate synthase |
| FTT0935c | FTL1274 | *bioC* | +2.0 | +1.7 | Biotin synthesis protein |
| FTT0933 | FTL1276 |  | +2.3 | +2.0 | Bifunctional protein, pseudogene |
| FTT0928c | FTL1282 |  | +2.2 | +2.5 | Beta-glucosidase-related glycosidase |
| FTT0735 | FTL1378/FTL1379 |  | +2.3 | +2.6 | Racemase/muconate lactonizing enzyme family protein, pseudogene |
| FTT1474c | FTL1395 | *galP1* | +2.1 | +2.2 | Galactose-proton symporter |
| FTT0800 | FTL1421 |  | +2.2 | +2.0 | Haloacid dehalogenase-like hydrolase family protein |
| FTT0776c | FTL1449 | *rnd* | +2.3 | +3.1 | Ribonuclease D |
| FTT1324 | FTL1485 |  | -2.5 | -2.5 | Hypothetical protein |
| FTT0721c | FTL1504 | *katG* | +2.7 | +2.2 | Peroxidase/catalase |
| FTT0728 | FTL1513 | *ybhF* | +2.1 | +2.0 | ABC transporter |
| FTT0512 | FTL1545 |  | -2.9 | -3.2 | SNO glutamine amidotransferase family protein |
| FTT0511 | FTL1546 |  | -2.1 | -3.0 | Pyridoxine biosynthesis protein |
| FTT0488c | FTL1574 |  | +2.5 | +1.7 | Major facilitator superfamily (MFS) transport protein |
| FTT0465 | FTL1599 |  | +2.1 | +1.9 | Hypothetical protein |
| FTT0464 | FTL1600 | *ansB* | +3.0 | +1.5 | Periplasmic L-asparaginase II precursor |
| FTT1622c | FTL1690 |  | +2.0 | +1.9 | Hypothetical membrane protein |
| FTT1623c | FTL1691 |  | +2.2 | +1.4 | Hypothetical protein |
| FTT0092c | FTL1763/FTL1764 | *appC* | -2.9 | -3.1 | Conserved hypothetical protein/Cytochrome oxidase bd-II, subunit I, pseudogene |
| FTT0083 | FTL1776 |  | +2.4 | +1.3 | Hypothetical membrane protein |
| FTT0070c | FTL1790 | *ampG* | -3.8 | -4.3 | Major facilitator superfamily (MFS) transport protein |
| FTT0029c | FTL1832 |  | -3.4 | -3.1 | Hypothetical protein |
| FTT0027c | FTL1834 | *lysA* | -2.1 | -2.0 | Diaminopimelate decarboxylase |
| FTT0026c | FTL1835 |  | -2.2 | -2.6 | Hypothetical protein |
| FTT1734c | FTL1876 | *fopA1* | -3.3 | -4.0 | Outer membrane protein fragment |
| FTT0178c | FTL1917 | *rimK* | -2.0 | -2.0 | 30S ribosomal protein S6 modification protein |
| FTT1783 | FTL1948 |  | +2.6 | +3.0 | Major facilitator superfamily (MFS) transport protein |
| FTT0718 | FTT1517 |  | +2.4 | +1.7 | Hypothetical protein |

Only those genes whose expression changes by a factor of 2 or more, with a *p*–value < 0.01, in either the Δ*mglA* or Δ*sspA* mutant background are listed. Negative values indicate genes that are positively regulated by MglA or SspA, whereas positive values indicate genes that are negatively regulated. LVS ORFs are referred to by the LVS (FTL number) and Schu S4 (FTT number) locus tags for convenience. Gene names and predicted products are as annotated by Entrez Gene at the NCBI.
